# Association of Cat Sensitization With Comorbid Asthma in Patients With Allergic Rhinitis: A Real‐World Study

**DOI:** 10.1002/wjo2.70113

**Published:** 2026-05-12

**Authors:** Wei‐Hao Wang, Xin Luo, Zhen‐Hao Xiao, Hong‐Kai Wang, Ya‐Na Zhang, Qin‐Tai Yang

**Affiliations:** ^1^ Department of Otolaryngology‐Head and Neck Surgery The Third Affiliated Hospital of Sun Yat‐Sen University Guangzhou China; ^2^ Department of Allergy The Third Affiliated Hospital of Sun Yat‐Sen University Guangzhou China; ^3^ Key Laboratory of Airway Inflammatory Disease Research and Innovative Technology Translation Guangzhou China

**Keywords:** allergic comorbidity, allergic rhinitis, asthma, cat ownership, cat sensitization, HDM

## Abstract

**Background:**

The prevalence of allergic rhinitis (AR) and asthma (AS) comorbidity (AR + AS) is increasing and is associated with severe allergic symptoms. The impact of cat dander sensitization on AR + AS remains unclear.

**Methods:**

In this retrospective study, 1154 patients with AR alone and 213 patients with AR + AS were enrolled at the Third Affiliated Hospital of Sun Yat‐Sen University from January 2021 to February 2024. Allergens in serum were measured using UniCAP system. Multivariate logistic regression was performed to assess risk factors of AR + AS.

**Results:**

Although family history of AR and AS, VAS (> 3), polysensitization, cat ownership, cat sIgE level, and age < 18 were associated with AR + AS, cat dander sensitization remained the prominent factor (OR = 3.743, *p* < 0.001). The positive rate of cat dander was higher in AR + AS group than AR alone group (25.4% vs. 10.5%, *p* < 0.001). The prevalence of AR + AS was the highest in the HDM+Cat+ group (33.8%). Moreover, the prevalence of cat‐sensitized AR + AS increased from 14.3% to 37.3% with elevated HDM sIgE levels. Co‐sensitization of HDM and cat dander (OR = 3.244, *p* = 0.036) and the cat ownership among cat‐sensitized patients (OR = 5.531, *p* < 0.001) were associated with AR + AS. However, in children, cat sensitization without ownership was associated with AR + AS, whereas this was not observed in adults.

**Conclusions:**

Cat sensitization is associated with AR + AS. Co‐sensitization to HDM and cat dander and cat ownership further increase the prevalence of AR + AS among the cat‐sensitized patients. Early detection of cat dander component sensitization may help identify at‐risk individuals and optimize management of AR + AS.

## Summary

- Cat dander sensitization was associated with concomitant asthma in allergic rhinitis.
- Co‐sensitization to house dust mite and cat dander further increased the occurrence of AR with asthma, and this proportion rose with increasing HDM sIgE levels.
- Cat ownership enhanced the association between cat dander sensitization and asthma comorbidity, whereas in children, sensitization without ownership remained relevant.

## 1 Introduction

Allergic rhinitis (AR) is a common upper airway condition and affects approximately 20% of the global population [1]. AR is known to be linked with other allergic disorders, such as asthma (AS), called AR comorbidity [2]. Based on differences in clinical symptoms, immunological mechanisms, and gene expression, AR with AS comorbidity (AR + AS) is considered distinct from AR alone [3, 4].

Increasing clinical evidence suggests that the rise in allergic comorbidity is linked to increased pet keeping [5]. Pets such as cats and dogs are an important source of indoor airborne allergens, contributing to the elevated prevalence of AR and more severe clinical presentation [6]. Cats are the pets most commonly implicated in the etiology of AR and AS. An international survey of over 27000 participants from 22 countries estimated that 57% of people have at least one pet, most commonly dogs (33%) and cats (23%) [7]. Although the prevalence of dog ownership is higher, cat sensitization is more common among allergic individuals [8, 9]. A retrospective study conducted in Beijing, China, found that sensitization to cats increased annually from 2017 to 2023, with 26.6% of surveyed individuals exhibiting cat sensitivity [6]. Consistently, our previous research reported that the prevalence of pet allergen sensitization among AR patients in Guangzhou was 23.4% and increased at an annual rate of 1.3% [10]. The impact of cat allergy on the development of allergic diseases, including allergic comorbidity, should be taken seriously, as its prevalence is increasing over time.

The relationship between the development of allergic disorders and cat allergy remains controversial [11]. A meta‐analysis of more than 77000 children from the European Union Child Cohort Network indicated that the early‐life cat ownership was not associated with AS development and cat‐specific sensitization [12]. Another study found that cat ownership had a significant bearing on the occurrence of childhood AS [13]. Conversely, Taniguchi and Kobayashi [14] found that cat ownership and exposure had negative effects on AS. Since AR alone and AR + AS are two distinct diseases, and risk factors for AR + AS are likely distinct from those for AR alone or AS alone. However, few studies focus on the difference of cat allergy between AR alone and AR + AS to elucidate the underlying mechanism of the onset of AR comorbidity.

In the current study, we investigated the sensitization of cat allergens in AR + AS prevalence. Our findings identify cat sensitization and cat ownership as the predictive factors for AR + AS, offering novel insight for risk stratification and guiding targeted clinical management in patients with AR with AS comorbidity.

## 2 Materials and Methods

### 2.1 Study Population

A total of 2980 patients with AR were enrolled at the Third Affiliated Hospital of Sun Yat‐Sen University from January 2021 to February 2024. This study was approved by the Ethics Committee (Ⅱ2024‐033‐01), and written informed consent was obtained from all participants.

AR, AS, atopic dermatitis (AD), and allergic conjunctivitis (AC) were diagnosed by clinical allergists based on patients' symptoms, clinical physical examination, and serum allergen‐specific immunoglobulin E (sIgE) test results according to the international consensus of diagnostics or expert consensus [15, 16, 17, 18, 19]. The diagnosis of adult asthma was based on pulmonary function tests and a prior asthma diagnosis by pulmonologists. For children, especially those younger than 6 who were unable to complete pulmonary function tests, the diagnosis of asthma was based on a prior asthma diagnosis by pediatricians and parent‐reported asthma.

**Figure 1:**
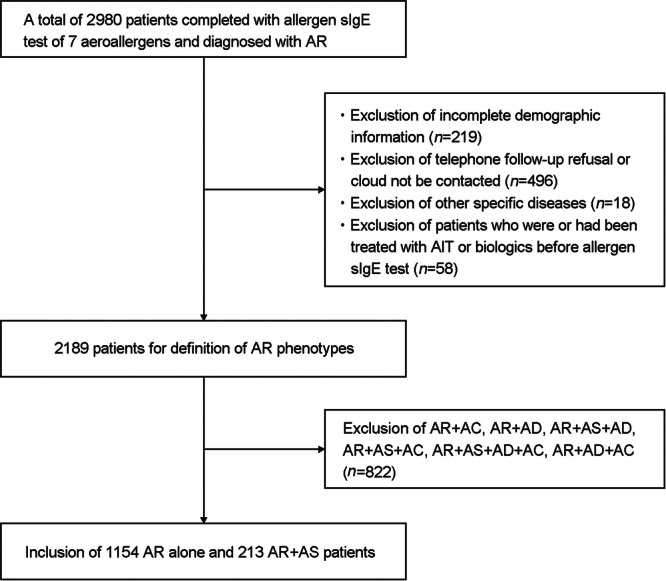
Flowchart for inclusion of patients.

Exclusion criteria for this study were (1) patients with incomplete demographic information (*n* = 219); (2) patients who refused to complete the questionnaire (*n* = 496); (3) patients with other diseases (parasitic infections/deep fungal infections/immunodeficiency diseases/malignant tumors) (*n* = 18); (4) allergen immunotherapy (AIT) or biologics before sIgE testing (*n* = 58). Ultimately, 2189 patients were included for AR phenotypes classification (Figure 1).

### 2.2 Assessment of Allergen‐Specific IgE

Allergen sIgE levels in serum were measured in all recruited patients. The UniCAP (Pharmacia Diagnostics, Uppsala, Sweden) instrument platform was used to determine the serum allergen‐sIgE of seven inhalant allergens including house dust mites (HDM), cat dander, dog dander, cockroaches, mold mixtures (*Candida/Penicillium notatum*/*Cladosporium*/*Alternaria*/*Aspergillus niger*), tree pollen combinations (elm/willow/poplar), grass pollen combination (ragweed/*artemisia argy*/*humulus*/*chenopodium*). Serum allergen sIgE levels were graded from level 0 to level 6 as follows: level 0 (0–0.34 IU/ml), level 1 (0.35–0.69 IU/ml), level 2 (0.70–3.49 IU/ml), level 3 (3.5–17.49 IU/ml), level 4 (17.5–49.9 IU/ml), level 5 (50.0–100 IU/ml) and level 6 (> 100 IU/ml). Tests with sIgE levels greater than or equal to 0.35 IU/ml were defined as sIgE positive. For patients who underwent multiple sIgE tests, only the first results were included.

### 2.3 Clinical Data

Information on the age, gender, body mass index (BMI, kg/m^2^), smoking status, family history of AR or AS, AR duration (classified as persistent: symptoms appearing more than 4 days per week and for more than 4 weeks; intermittent: symptoms appearing for less than 4 days a week or for less than 4 weeks) [15] and the nasal total visual analogue scale (VAS) scores were collected. In multivariate logistic regression model, the VAS scores were classified into ≤ 3 (mild group), and > 3 group (moderate‐severe group) [20]. The age was classified into < 18 years as children and ≥ 18 years as adults. Information on current pet ownership was also collected for the analysis on the association with the different AR phenotypes.

### 2.4 Definition of AR Phenotypes

AR phenotypes were classified into (*n* = 2189):

AR alone (*n* = 1154): Patients with AR who did not have AS, AD, and AC.

AR multimorbidity (*n* = 1035): AR patients with more than one additional allergic condition. AR multimorbidity includes AR + AS, AR + AC, AR + AD, AR + AS + AD, AR + AS + AC, AR + AD + AC, and AR + AS + AD + AC. In order to specifically clarify the cat sensitization to AR alone and AR + AS and minimized confounding from other allergic diseases, the present study focused on patients only with asthma comorbidity (*n* = 213) (Figure 1).

### 2.5 Statistical Analysis

Statistical analyses were conducted using SPSS version 23 (SPSS, Chicago, IL, USA). Continuous data with non‐normal distribution were described using the median (interquartile range [IQR]). The Mann–Whitney *U* test was used to compare the difference of numerical data distribution between groups. The chi‐square test and Bonferroni correction were used for comparisons between categorical groups. Multivariate logistic regression was applied to assess the association between predictive variables and AR + AS development. Odds ratios (OR) were adjusted for gender, age, VAS scores, family history of AR, family history of AS, HDM sensitization, cat sensitization, cat ownership, and polysensitization in different models. The OR derived from the logistic regression analyses were accompanied by their respective 95% confidence intervals (95% CI). Statistical significance was declared when the *p* value was < 0.05.

## 3 Results

### 3.1 Demographics and Baseline Characteristics

A total of 1367 patients with AR were included in the current analysis, comprising 1154 patients with AR alone (84.4%) and 213 patients with AR + AS (15.6%). The demographic and clinical characteristics of the study population were presented in Table 1. AR + AS patients were younger (11.0 [9.0, 16.0]) than patients with AR alone (17.0 [10.0, 32.0]) (*p* < 0.001). AR + AS was more common in the children group (age < 18 years old) than in the adult group (*p* < 0.001). The total nasal VAS scores were utilized to assess the severity of AR we found AR + AS patients suffered from a more severe rhinitis (*p* < 0.001).

**Table 1:** Demographic features of AR alone patients and AR + AS patients.

	Total sample (*n* = 1367)	AR alone (*n* = 1154)	AR + AS (*n* = 213)	*p* value*
Gender (male), *n* (%)	753 (55.1)	628 (54.4)	125 (58.7)	0.250
Age (years), median (*P*_25_, *P*_75_)	15.0 (10.0, 31.0)	17.0 (10.0, 32.0)	11.0 (9.0, 16.0)	< 0.001
< 18 years old, *n* (%)	746 (54.6)	583 (50.5)	163 (76.5)	< 0.001
≥ 18 years old, *n* (%)	621 (45.4)	571 (49.5)	50 (23.5)	
Rhinitis duration				0.316
Intermittent, *n* (%)	926 (67.7)	788 (68.3)	138 (64.8)	
Persistent, *n* (%)	441 (32.3)	366 (31.7)	75 (35.2)	
Total nasal VAS scores, median (*P*_25_, *P*_75_)	3.0 (2.0, 4.0)	2.0 (2.0, 4.0)	4.0 (3.0, 4.0)	< 0.001
≤ 3, *n* (%)	956 (69.9)	857 (74.3)	99 (46.5)	< 0.001
> 3, *n* (%)	411 (30.1)	297 (25.7)	114 (53.5)	
Overweight/obesity by BMI, *n* (%)	80 (5.9)	65 (5.6)	15 (7.0)	0.421
Tobacco status, *n* (%)				0.214
Never smoker	1265 (92.5)	1063 (92.1)	202 (94.8)	
Ex‐smokers	56 (4.1)	48 (4.2)	8 (3.8)	
Current smoker	46 (3.4)	43 (3.7)	3 (1.4)	
Family history of AS, *n* (%)	84 (6.1)	54 (4.7)	30 (14.1)	< 0.001
Family history of AR, *n* (%)	288 (21.1)	219 (19)	69 (32.4)	< 0.001
Current cat ownership, *n* (%)	129 (9.4)	93 (8.1)	36 (16.9)	< 0.001
Polysensitization, *n* (%)^a^	366 (26.8)	291 (25.2)	75 (35.2)	0.002

Abbreviations: AR, allergic rhinitis; AR + AS, AR and asthma comorbidity; BMI, body mass index; VAS, visual analogue scale.

* *p* values for Mann–Whitney *U* test for continuous variables or chi‐square test for categorical variables.

a Polysensitization represents sensitization to more than 1 inhalant allergen.

**Figure 2:**
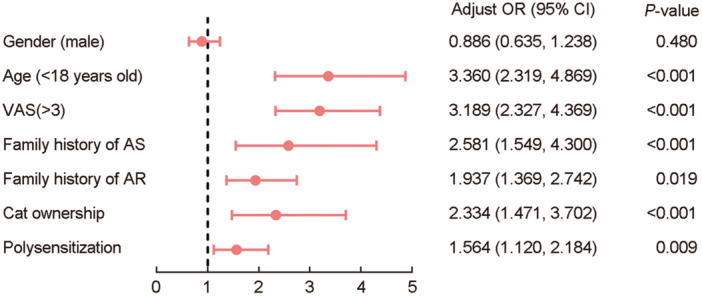
Analysis of risk factors for AR + AS. Odds ratios for AR + AS were multifactorial and adjusted for gender, age, VAS scores, family history of AR, family history of AS, cat ownership, and polysensitization. AR + AS, allergic rhinitis and asthma comorbidity; CI, confidence interval; OR, odds ratios; VAS, visual analogue scale.

Multivariate logistic regression was applied and found that AR + AS was significantly associated with the family history of AR (OR = 1.937; 95% CI: 1.369, 2.742; *p* = 0.019), the family history of AS (OR = 2.581; 95% CI: 1.549, 4.300; *p* < 0.001), VAS scores (> 3) (OR = 3.189; 95% CI: 2.327, 4.369; *p* < 0.001), polysensitization (OR = 1.564; 95% CI: 1.120, 2.184; *p* = 0.009), cat ownership (OR = 2.334; 95% CI: 1.471, 3.702; *p* < 0.001) and children (age < 18 years old) (OR = 3.360; 95% CI: 2.319, 4.869; *p* < 0.001) (Figure 2).

### 3.2 The Association Between Allergen Sensitization and AR + AS

The present study conducted an allergen sIgE test for seven aeroallergens. The results showed that HDM was still the most common inhalant allergen among patients with AR alone (92.2%) and AR + AS patients (94.4%) (Figure S1). The HDM sIgE concentration in AR + AS patients was higher than that in AR alone group (*p* = 0.002, Table S1), suggesting that elevated HDM sIgE levels were associated with more severe allergic reactions. We found that there were no significant differences in the positive rates of mold mixtures, dog dander, cockroaches, tree pollen combinations, and grass pollen combinations between these two groups (*p* > 0.05, Figure S1). Interestingly, only the cat dander sensitization showed significantly different distribution between AR alone patients (10.5%) and AR + AS patients (25.4%, *p* < 0.001, Figure S1).

Furthermore, the multivariate logistic regression was employed to assess the association between those seven allergens mentioned above and AR + AS development. As seen in Table 2, cat dander sensitization (OR = 3.743; 95% CI: 1.812, 7.729; *p* < 0.001) was significantly associated with the occurrence of AR + AS instead of HDM, mold mixtures, dog, cockroaches, grass pollen, and tree pollen sensitization. Similarly, cat sIgE levels were significantly associated with AR + AS (OR = 1.763; 95% CI: 1.428, 2.177; *p* < 0.001) (Table S2), which indicates that cat allergy might be a risk factor for AR + AS prevalence.

**Table 2:** Multivariate logistic analysis of association between aeroallergen sensitization and AR + AS.

Allergens	AR + AS	
	OR (95% CI)^a^	*p* value
HDM (positive)	2.049 (0.859, 4.889)	0.106
Cat dander (positive)	3.743 (1.812, 7.729)	< 0.001
Mold mixture (positive)	1.294 (0.565, 2.965)	0.543
Dog dander (positive)	1.140 (0.473, 2.750)	0.770
Cockroaches (positive)	1.536 (0.718, 3.288)	0.269
Tree pollens (positive)	2.020 (0.674, 6.052)	0.209
Grass pollens (positive)	1.742 (0.682, 4.450)	0.246

Abbreviations: AR + AS, AR and asthma comorbidity; CI, confidence Interval; HDM, house dust mites; OR, odds ratios.

^a^ Adjust for VAS scores, family history of AR, family history of AS, age, gender, polysensitization, cat ownership, and whether other allergen sensitization or not.

### 3.3 The Effects of Polysensitized to HDM and Cat Dander on the Prevalence of AR + AS

**Figure 3:**
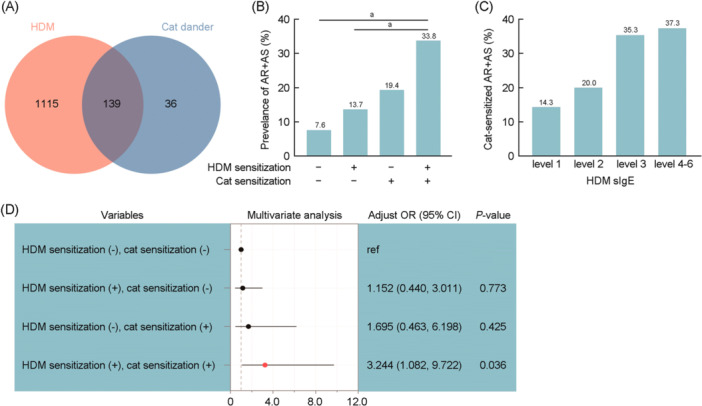
Impacts of HDM on the cat‐sensitized AR + AS prevalence. (A) The proportion of cat sensitization in HDM‐sensitized AR patients. 79.4% of cat‐sensitized patients co‐sensitized to HDM. (B) The prevalence of AR + AS in different sensitization pattern groups of HDM and cat dander. (C) The prevalence of cat‐sensitized AR + AS in different HDM sIgE levels groups. (D) Adjusted association between co‐sensitization of HDM and cat dander and AR + AS prevalence. Odds ratios for each group were multifactorial and adjusted for gender, age, VAS scores, family history of AR, family history of AS, cat ownership and polysensitization. ^a^ *p* < 0.001. AR, allergic rhinitis; AR + AS, allergic rhinitis and asthma comorbidity; CI, confidence interval; HDM, house dust mites; OR, odds ratios.

HDM is the most common inhalant allergen among patients with AR + AS in South China. In our study, 79.4% (139/175, Figure 3A) of cat‐sensitized AR patients were co‐sensitized to HDM, which was more common in the AR + AS subgroup (87.0%, 47/54). Cat dander co‐sensitization with HDM was the main allergen pattern among AR + AS patients. In order to clarify the relation of HDM sensitization and cat dander sensitization on the AR + AS prevalence, AR patients were categorized into 4 subgroups as follows: both negative to HDM and cat dander sensitization (HDM‐Cat−), HDM‐positive sensitization and cat dander‐negative sensitization (HDM+Cat−), HDM‐negative sensitization and cat dander‐positive sensitization (HDM‐Cat +), co‐positive to HDM and cat dander sensitization (HDM+Cat +). The prevalence of AR + AS was highest in the HDM+Cat+ group (33.8%) and higher than in HDM+Cat− group (13.7%, *p* < 0.001, Figure 3B), suggesting that sensitization to cat allergen might increase the risk of AR + AS prevalence. Moreover, the prevalence of cat‐sensitized AR + AS increased from 14.3% to 37.3% with HDM sIgE levels (Figure 3C), suggesting that co‐sensitization to HDM and cat dander exacerbated the occurrence of AR + AS. We further analyzed the association between HDM and cat dander sensitization and AR + AS. Compared to the baseline (HDM‐Cat‐group), AR + AS was only significantly associated with the co‐sensitization with HDM and cat dander (OR = 3.244; 95% CI: 1.082, 9.722; *p* = 0.036, Figure 3D). These results indicated that cat dander sensitization is associated with the prevalence of AR + AS, and that co‐sensitization to HDM may further increase its incidence.

### 3.4 The Impact of Cat Ownership on the Cat‐Sensitized AR + AS Prevalence

**Figure 4:**
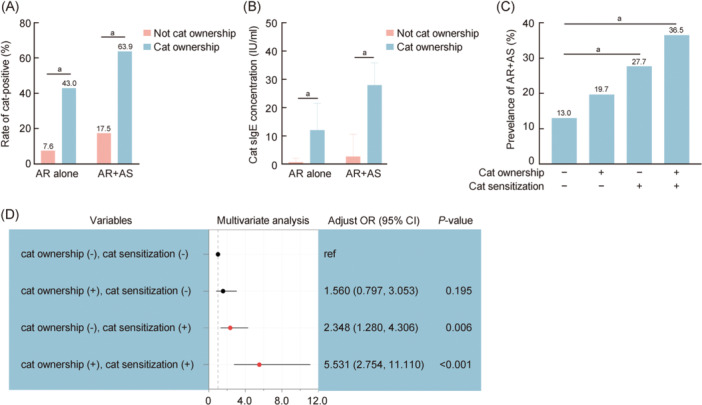
Impacts of cat ownership on the cat‐sensitized AR + AS prevalence. (A) The positive rate of cat dander sIgE sensitization in different cat ownership groups among AR patients. (B) The cat sIgE concentration in different cat ownership groups among AR patients. (C) The prevalence of AR + AS in different cat ownership and cat dander sensitization status. (D) Adjusted association between cat ownership and cat dander and AR + AS prevalence. Odds ratios for each group were multifactorial and adjusted for gender, age, VAS scores, family history of AR, family history of AS, HDM sensitization and polysensitization. ^a^ *p* < 0.001. AR, allergic rhinitis; AR + AS, allergic rhinitis and asthma comorbidity; CI, confidence interval; OR, odds ratios.

Among the main factors related to the cat sensitization, the role of cat ownership was still controversial. We found that the cat ownership was more common in patients with AR + AS compared to AR alone patients (16.9% vs. 8.1%, *p* < 0.001, Table 1). Moreover, keeping the cat could increase the positive rate of cat dander sensitization as well as the sIgE concentration in AR + AS patients (*p* < 0.001, Figure 4A,B). Cat ownership was related to a higher cat sIgE‐positive rate and stronger allergic response. For further distinguishing the effect of cat ownership on the prevalence of cat‐sensitized AR + AS, we categorized the AR patients into 4 groups as follows: not owing a cat and cat dander‐negative sensitization (Ownership‐Sensitization−), owning a cat and cat dander‐negative sensitization (Ownership+Sensitization−), not owing a cat and cat dander‐positive sensitization (Ownership‐Sensitization + ), owning a cat and cat dander‐positive sensitization (Ownership+Sensitization + ). The prevalence of AR + AS was the highest in the Ownership+Sensitization+ group (36.5%, Figure 4C).

We analyzed the model with different cat ownership and cat dander sensitization status as the variables and the occurrence of AR + AS as the outcome. Cat sensitization was associated with the risk of AR + AS development, even in the absence of cat ownership (OR = 2.348; 95% CI: 1.280, 4.306; *p* = 0.006, Figure 4D). Cat ownership further increased the risk of AR + AS in cat‐sensitized patients (OR = 5.531; 95% CI: 2.754, 11.10; *p* < 0.001; Figure 4D).

### 3.5 Association Between Cat Ownership and Cat‐Sensitized AR + AS in Children Differ From Those in Adults

We stratified AR patients into children group (< 18 years old) and adults (≥ 18 years old), and found that cat ownership showed different effects on the AR + AS prevalence among cat‐sensitized patients (Table 3). In children group, cat ownership and sensitization was significantly associated with the AR + AS (OR = 4.147; 95% CI: 1.535, 11.202; *p* = 0.005). Interestingly, cat sensitization was still associated with AR + AS development among patients without cat ownership (OR = 2.634; 95% CI: 1.297, 5.348; *p* = 0.007), suggesting the importance of cat sensitization in the development of AR + AS among children. However, in adult group, only the combination of owning cats and cat sensitization was significantly associated with the risk of AR + AS development (OR = 6.170; 95% CI: 2.211, 17.222; *p* < 0.001), suggesting that cat ownership exacerbated the occurrence of AR + AS among adult cat‐sensitized patients. The different effects of cat ownership on the AR + AS development between children and adult cat‐sensitized AR patients demonstrated that cat allergen exposure routes (direct or indirect), airborne cat allergen concentration, and age‐dependent severity of sIgE responses to cat allergens influenced AR + AS development.

**Table 3:** Cat sensitization and ownership in different age groups.

Ownership and sensitization status		Children		Adults	
Cat ownership	Cat sensitization	OR (95% CI)^a^	*p* value	OR (95% CI)^a^	*p* value
(−)	(−)	1 (reference)		1 (reference)	
(+)	(−)	1.288 (0.578, 2.870)	0.536	2.497 (0.732, 8.516)	0.144
(−)	(+)	2.634 (1.297, 5.348)	0.007	1.250 (0.330, 4.732)	0.743
(+)	(+)	4.147 (1.535,11.202)	0.005	6.170 (2.211, 17.222)	< 0.001

*Note:* (−) represents not owning a cat or cat dander‐negative sensitization; (+) represents owning a cat or cat dander‐positive sensitization.

Abbreviations: AR + AS, AR and asthma comorbidity; CI, confidence interval; OR, odds ratio.

^a^ Adjusted for gender, age, VAS scores, family history of AR, family history of AS, house dust mite sensitization, and polysensitization.

## 4 Discussion

In this study, we explored the cat dander allergy pattern in patients with AR alone versus those with AR and asthma comorbidity and demonstrated that cat sensitization is a major contributing factor to the development of AR and asthma comorbidity. In addition, co‐sensitization to HDM and cat dander, as well as cat ownership, may potentiate the risk of developing AR and asthma comorbidity. To the best of our knowledge, this is the first study to concurrently evaluate cat sensitization with HDM sensitization and cat ownership to explore their interrelationships in the development of AR and asthma comorbidity in South China.

Previous studies have demonstrated that cat sensitization increases the risk of developing AR alone and asthma alone in both children [6, 21] and adults [22]. In the current study, we found that cat sensitization was more common in patients with AR and asthma comorbidity than in those with AR alone. Furthermore, elevated cat dander sIgE levels were observed in patients with AR and asthma comorbidity compared with those with AR alone. Our results underscore their potential utility as a predictor of AR and asthma comorbidity. Consistently, retrospective study enrolled 1393 children found that the cat pelts had the significantly highest prevalence in children with comorbid AR and asthma rather than AR alone and asthma alone [23]. Furthermore, exposure to cat allergens has been demonstrated to be associated with an increased prevalence of asthma exacerbations [6]. In addition, after exposure to cat allergens, 67% and 52% of participants observed early asthma reactions after the first and second exposure [24]. However, a cross‐sectional study of 3739 patients with AR conducted in eastern China found sensitization to *Aspergillus fumigatus*, *D. pteronyssinus* and dog dander, rather than cat dander, was a risk factor for asthma and AR comorbidity [25]. This discrepancy may be attributed to regional differences in allergen exposure and variations in patient populations.

The MeDALL (Mechanisms of the Development of Allergy) provides new insights into the links between polysensitization and allergic multimorbidity [3]. Polysensitization is associated with stronger global IgE responses, more complex disease phenotypes, and more severe symptoms. In the current study, higher prevalence of allergen polysensitization was observed in patients with AR and asthma comorbidity than those with AR alone. Additionally, allergen polysensitization was associated with AR and asthma comorbidity. A study investigating the impact of *Dermatophagoides pteronyssinus* sensitization on grass pollen found that the risk of asthma increased significantly with higher *Dermatophagoides pteronyssinus* sIgE levels [26]. However, the influence of HDM sensitization on the prevalence of cat dander‐sensitized AR and AS comorbidity has been understudied. HDM is the most common indoor aeroallergen among AR patients in South China, and polysensitization to HDM and cat dander is ubiquitous in AR multimorbidity [27, 28]. Moreover, polysensitization combined with HDM and animal allergen components such as *Canis familiaris* allergen 4 (Can f 4) and Fel d 1, was identified as a risk factor for severe allergic symptoms [29]. In the present study, AR patients were classified according to HDM and cat dander sensitization patterns to evaluate their effects in polysensitized individuals. Of note, we demonstrated that although HDM positivity rate reached 92.2%–94.4%, the prevalence of AR + AS was lower (13.7%) than that in “HDM+Cat + ” group (33.8%), suggesting cat dander may play an important role in promoting the development of asthma in sensitized individuals, although this requires further experimental validation.

The relationship between cat ownership and cat dander allergen sensitization remains controversial [11]. A study found a negative association between having a cat at home and a positive skin prick test response to cat [30]. Households with cats are a major source of cat allergens, and Fel d 1 is highly prevalent, with a positive rate of 91.67% among cat‐sensitized patients [24, 31].

Fel d 1 is a heat‐stable protein, resistant to degradation, and particles measuring approximately 2.5–5 μm can remain suspended in indoor air, easily inhaled into the nasal cavity and reach the lower respiratory tract, leading to airway hyperresponsiveness [32]. Cat allergens can persist for 6–9 months even after pet removal, highlighting the prolonged risk of exposure [33]. Direct exposure to cat allergen has been associated with poor control of AR symptoms in cat owners and an increased prevalence of cat allergy among children following exposure [24, 34]. Moreover, acquiring a cat in adulthood almost doubles the risk of developing new‐onset cat sensitization [35]. Consistently, in our study, cat ownership was observed with an increased positive rate of sIgE to cat dander and higher cat dander sIgE levels. Collectively, these findings indicate that cat ownership might increase the risk of both direct allergen exposure and subsequent cat dander sensitization.

Next, stratifying patients by age revealed differences in sensitization patterns, with cat‐sensitized children exhibiting a higher risk of AR and asthma comorbidity than adults, even in the absence of cat ownership. Studies have shown that cat allergen exposure is common in schools, hospitals and day care centers, and that elevated exposure levels in these settings can exceed those found in homes [36, 37]. Thus, cat‐sensitized children without household cats or direct contact can still be exposed to sufficient allergens to trigger symptoms. While both children and adults can be exposed to high levels of cat allergens in cat‐free settings, the differences in AS comorbidity outcomes may be explained by several factors: (1) children have higher respiratory rate [38] and lower nasal deposition efficiency of air particles [39, 40], allowing more allergens to reach the lower respiratory tract and potentially trigger AS symptoms and (2) children's immature immune systems and reduced tolerance result in enhanced IgE‐mediated reactivity, particularly to cat dander. In contrast, adults possess more mature immune systems with stronger immunotolerance, requiring higher allergen exposure to elicit respiratory symptoms.

There are some limitations in the present study. First, pulmonary function tests were not performed in all AR patients to exclude the presence of potential asthma, which may have led to an underestimation of the AR + AS population. In addition, the current study is retrospective, single‐center, and cross‐sectional, which limits the ability to establish a causal relationship between cat sensitivity and AR + AS comorbidity. Second, detailed information such as the timing of cat ownership (prenatal, early childhood, puberty, or adulthood), number of cats owned, duration of contact with pets, pet allergen exposure in schools, relatives' homes, or other public environments, and the age of onset of allergic symptoms is lacking. Therefore, the present study was unable to further analyze the association between detailed cat exposure patterns and cat sensitization, which might lead to the misunderstanding of the true impact of cat sensitization. Third, although the specific age distribution, gender composition and asthma severity were not provided in the current study, we found that he prevalence of comorbid asthma in children with AR was higher than that in AR adults, which is consistent with previous studies, showing that 30% of children with AR have asthma, compared with 10.2% of adults with AR [41, 42, 43]. Due to these limitations, multicenter, large‐scale longitudinal cohort studies incorporating detailed patient data such as pulmonary function tests, age distribution, and gender composition are needed.

## 5 Conclusions

In conclusion, the current study demonstrated that sensitization to cat dander was associated with AR and AS comorbidity in South China. In addition, cat and HDM co‐sensitization and cat ownership could result in an increased prevalence of AR with AS comorbidity. A future prospective study conducted by the detection of sensitization to cat dander components will allow for the screening of individuals at risk of developing AR with AS comorbidity, thereby optimizing the management of respiratory allergic diseases in this subgroup of patients.

## Author Contributions

Wei‐Hao Wang and Xin Luo collected patient data, conducted statistical analyses, and drafted the manuscript. Zhen‐Hao Xiao and Hong‐Kai Wang reviewed the data and revised the tables and manuscript. Ya‐Na Zhang and Qin‐Tai Yang proposed the manuscript concept and took charge of the study design, project supervision, and final approval of the version.

## Ethics Statement

This study was approved by the Ethics Committee for Human Study at the Third Affiliated Hospital of Sun Yat‐Sen University (China), and all subjects provided informed consent.

## Conflicts of Interest

The authors declare no conflicts of interest.

## Supporting information


Supporting File 1

Supporting File 2

## Data Availability

The data that support the findings of this study are available from the corresponding author upon reasonable request.
