# Supplementary material for: Association of Cat Sensitization With Comorbid Asthma in Patients With Allergic Rhinitis: A Real‐World Study
Source: World J Otorhinolaryngol Head Neck Surg. 2026 May 12:10.1002/wjo2.70113. Online ahead of print. doi: 10.1002/wjo2.70113 (PMC13398456; doi:10.1002/wjo2.70113)
Supplement: Supplementary file 1 — Supporting File 1 [file WJO2-9999-0-s001.docx]

**Supplementary for the article entitled “Association of cat sensitization with comorbid asthma in patients with allergic rhinitis: a real-world study”**

| **Table S1** Allergen sIgE concentration bewteen AR and AR+AS. | | | |
| --- | --- | --- | --- |
|  | Allergen concentration (IU/mL, median [*P*_25_, *P*_75_]) | | |
| Inhalant allergens | AR alone | AR+AS | *P*-value^*^ |
| HDM | 21.2 (3.1, 60.0) | 34.6 (8.5, 67.0) | 0.002 |
| Cat dander | 1.2 (0.5, 8.0) | 12.7 (2.1, 27.0) | <0.001 |
| Mold mixture | 0.6 (0.4, 2.9) | 1.4 (0.4, 2.0) | 0.517 |
| Dog dander | 1.0 (0.5, 3.4) | 1.7 (0.7, 5.6) | 0.337 |
| Cockroaches | 0.5 (0.4, 1.2) | 1.7 (0.5, 4.3) | 0.018 |
| Tree pollens | 0.4 (0.4, 0.6) | 0.5 (0.4, 0.7) | 0.930 |
| Grass pollens | 0.5 (0.4, 1.8) | 0.4 (0.4, 0.5) | 0.332 |

Abbreviation: AR, allergic rhinitis; AR+AS, AR and asthma comorbidity; HDM, house dust mite;

sIgE, specific immunoglobulin E; CI, Confidence Interval; OR, odds ratio.

Note. ^*^*P*-values for Mann-Whitney U test for continuous variable.

**Table S2**. Multivariate logistic analysis of association between allergen sIgE and AR+AS.

|  |  | Odds of AR+AS | |
| --- | --- | --- | --- |
| Allergen sIgE levels^*^ |  | OR (95% CI)^§^ | *P*-value |
| HDM |  | 1.053 (0.945, 1.172) | 0.349 |
| Cat dander |  | 1.763 (1.428, 2.177) | ＜0.001 |
| Mold mixture |  | 1.034 (0.703, 1.521) | 0.865 |
| Dog dander |  | 0.966 (0.667, 1.399) | 0.855 |
| Cockroaches |  | 1.296 (0.939, 1.789) | 0.115 |
| Tree pollens |  | 1.275 (0.602, 2.697) | 0.526 |
| Grass pollens |  | 1.339 (0.809, 2.218) | 0.256 |

Abbreviation: AR+AS, AR and asthma comorbidity; HDM, house dust mites;

sIgE, specific immunoglobulin E; CI, Confidence Interval; OR, odds ratio.

Note.^*^The allergen sIgE were classified into levels 0 to 6.

^§^Adjusted for VAS scores, family history of AR, family history of AS, age, gender,

polysensitization, cat ownership and whether other allergens sensitization or not.

**Table S3**. The basic characteristics of “included/excluded”populations.

|  | Total population | Included population | Excluded population | *P*-value^*^ |
| --- | --- | --- | --- | --- |
|  | (n=2484) | (n=1367) | (n=1117) |  |
| Gender (male), n (%) | 1368 (55.1) | 753 (55.1) | 615 (55.1) | 0.990 |
| Age(years), median (*P*_25_, *P*_75_) | 15.0 (9.0, 30.0) | 15.0 (10.0, 31.0) | 14.0 (9.0, 29.0) | 0.531 |
| < 18 years old, n (%) | 1378 (55.5) | 746 (54.6) | 632 (56.6) | 0.316 |
| ≥ 18 years old, n (%) | 1106 (445) | 621 (45.4) | 485 (43.4) |  |
| Rhinitis duration |  |  |  | 0.149 |
| Intermittent, n (%) | 1652 (66.5) | 926 (67.7) | 726 (65.0) |  |
| Persistent, n (%) | 832 (33.2) | 441 (32.3) | 391 (35.0) |  |
| Total nasal VAS scores, median (*P*_25_, *P*_75_) | 3 (2.0, 4.0) | 3.0 (2.0, 4.0) | 3.0 (2.0, 4.0) | 0.990 |
| ≤ 3, n (%) | 1704 (68.6) | 956 (69.9) | 748 (67.0) | 0.113 |
| > 3, n (%) | 780 (31.4) | 411 (30.1) | 369 (33.0) |  |
| Overweight/Obesity by BMI, n (%) | 149 (6.0) | 80 (5.9) | 69 (6.2) | 0.734 |
| Tobacco status, n (%) |  |  |  | 0.678 |
| Never smoker | 2288 (92.1) | 1265 (92.5) | 1023 (91.6) |  |
| Ex-smokers | 107 (4.3) | 56 (4.1) | 51 (4.6) |  |
| Current smoker | 89 (3.6) | 46 (3.4) | 43 (3.8) |  |
| Family history of AS, n (%) | 164 (6.6%) | 84 (6.1) | 80 (7.2) | 0.310 |
| Family history of AR, n (%) | 553 (22.3) | 288 (21.1) | 265 (23.7) | 0.113 |
| Current cat ownership, n (%) | 246 (10.0) | 129 (9.4) | 117(10.5) | 0.389 |
| Polysensitization, n (%)^§^ | 1069 (43.0) | 366 (26.8) | 703 (62.9) | < 0.001 |

Abbreviation: VAS, Visual Analogue Scale; BMI, Body Mass Index.

Note. ^*^ *P*-values for Mann-Whitney U test for continuous variables or chi-square test for categorical variables.

^§^Polysensitization represents sensitization to more than 1 inhalant allergens.

**Figure legends**

Figure S1. Positive rate of allergen sIgE sensitization. AR alone group: allergen-positive cases/total number of AR alone (n=1154). AR+AS group: allergen-positive cases/total number of AR+AS (n=213). ^***^*P* < 0.001. AR, allergic rhinitis; AR+AS, allergic rhinitis and asthma comorbidity.
