# Supplementary figures and images for: Association of Cat Sensitization With Comorbid Asthma in Patients With Allergic Rhinitis: A Real‐World Study
Source: World J Otorhinolaryngol Head Neck Surg. 2026 May 12:10.1002/wjo2.70113. Online ahead of print. doi: 10.1002/wjo2.70113 (PMC13398456; doi:10.1002/wjo2.70113)

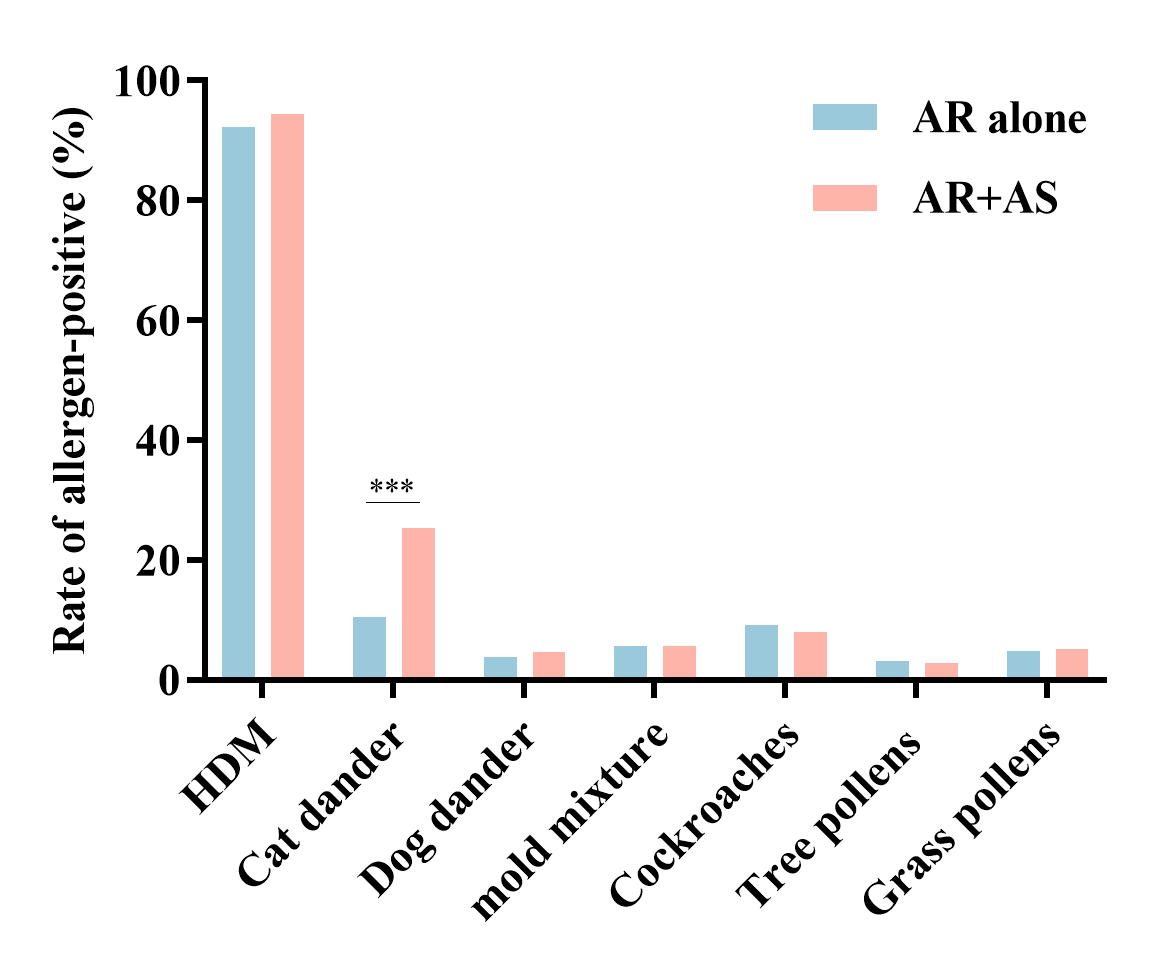

Supplement: Supplementary file 2 — Supporting File 2 [file WJO2-9999-0-s002.tif]
